# 

**Published:** 1887-04

**Authors:** 


### BOOKS RECEIVED.

*The Science and Art of Obstetrics.* By Theophilus Parvin, M.D., L.L.D

*Clinical Manual for the Study of Medical Cases.* By James Finlayson, M.D.

*Wear and Tear, or Hints for the Overworked.* By S. Weir Mitchell, M.D., L.L.D.

*On Diseases of the Lungs and Pleura, including Consumption.* By R. Douglas Powell, M.D.

*Hand-book of Practical Medicine. Vol. IV. Diseases of the Blood, and Nutrition and Infective Diseases.* By Dr. Hermann Eichhorst.

*A Clinical Manual of Diseases of the Ear.* By Laurence Turnbull, M.D., Ph.G.

*Diseases of the Joints.* By Howard Marsh, F.R.C.S.

*The Principles and Practice of Operative Surgery.* By Stephen Smith A.M.,.M.D.

*A Text-book on Surgery—General, Operative and Mechanical.* By John A. Wyeth, M.D.

*Transactions of the Association of American Physicians.* First session.

*Léçons sur les Maladies du Système Nerveux, faites á la Salpêtrière,* par J. M. Charcot.

*Collected Papers, Harvard Medical School, 1880–1886.*

*A Practical Treatise on Obstetrics.* By Dr. A. Charpentier. Translated by Egbert H. Grandin, M.D.

*Hand-book of Materia Medica, Pharmacy and Therapeutics, etc.* By Sam uel O. L. Potter, A.M., M.D.

*Diseases of Women. A Hand-book for Physicians and Students.* By Dr. F. Winckel. Translated under the supervision of Theophilus Parvin, M.D.

*On Fevers: their History, Etiology, Diagnosis and Treatment.* By Alexander Collie, M.D. (Aberd.)

*The Year-book of Treatment for 1886.*

*Refraction of the Eye. Its Diagnosis and Correction of Its Errors.* By A. Stanford Morton, M.B., F.R.C.S. (Ed.)

### REPORTS AND PAMPHLETS RECEIVED.

A Contribution to the Climatological Study of Phthisis in Pennsylvania. *By William Pepper, M.D., LL.D.*

Report of the Commission of the Southern Illinois Penitentiary, at Chester.

Report of the Board of Trustees of the Eastern Michigan Asylum, at Pontiac.

Researches into the Etiology of Dengue. *By J. W. McLaughlin, M.D.*

The Antiseptic Treatment of Summer Diarrhœa. *By L. Emmett Holt, A.M., M.D.*

Periostitis. *By N. Senn, M.D.*

Practical Observations on the Gonococcus and Roux's Method of Determining its Identity. *By Charles W. Allen, M.D.*

On a Mode of Determining, with the Prisoptometer, the Degree of Latent Hypermetropia without Mydriatics. *By H. Culbertson, M.D.*

Electrolysis in Gynæcology. *By Franklin H. Martin, M.D.*

Ueber Wirkung, therapeutischen Werth und Gebrauch des neuen Karlsbader Quellsalzes, nebst dessen Beziehung zum Karlsbader Thermalwasser. *Von Dr. W. Jaworski.*

Mittheilungen ueber das Ichthyol. *Von Dr. Ackermann, Weimar.*

Mittheilungen ueber Ichthyol. *Von Prof. Dr. Baumann, in Freiburg und Geheimrath von Nussbaum, in München.*

Notiz ueber das Ichthyol. *Von Prof. Dr. Ernst Schweninger.*

Ueber den Einfluss der Ichthyolpräparate auf den Stoffwechsel. *Von Prof. Dr. Zuelzer.*

Ichthyol und Resorein. *Von Dr. P. G. Unna.*

Ueber Erysipelas. *Von Geheimrath von Nussbaum.*

Sterility. *By Wm. H. Wathen, M.D.*

Report on the Diseases of the Rectum. *By Joseph M. Mathews, M.D.*

President's Address, Tenth Annual Meeting of the Detroit Medical and Library Association. *By C. J. Lundy, A.M., M.D.*

Report of the Surgeon-General of the Army to the Secretary of War, for the year ending June 30th, 1886.

Annual Report of the Supervising Surgeon-General of the Marine Hospital Service of the United States, for the fiscal year 1886.

An Epitome of the Newer Materia Medica Preparations. *By Parke, Davis & Company.*

Transactions of the American Ophthalmological Society. Twenty-second Annual Meeting, 1886.

An Address on the Relation of Quarantine to Shipping Interests. *By Joseph Holt, M.D.*

A Case of Pyelitis of Nineteen Years' Standing, caused by a Renal Calculus; Recovery. *By Augustus V. Park, M.D.*

A Case of Ante-Partum Hemorrhage at Term. Recovery. *By Augustus V. Park, M.D.*

The Relative Influence of Maternal and Wet-Nursing on Mother and Child. *By Joseph Edcil Winters, M.D.*

Laryngology and its Cognate Branches in America, and the Simple and Most Efficient Treatment of Diphtheria. *By W. H. Daly, M.D.*

On the Transmission of Syphilis, and on the Earliest Case of Whooping-Cough. *By Robert J. Lee, M.D., F.R.C.P.*

Three Cases of Fracture of the Skull. *By Oscar J. Coskery, M.D.*

The Curette as a Diagnostic and Therapeutic Agent in Gynæcology and Obstetrics. *By B. Bernard Browne, M.D.*

The Human Color-Sense considered as the Organic Responseto Natural Stimuli. *By L. Webster Fox, M.D., and George M. Gould, A.B.*

Notes on Hospital Practice in Vienna. *By Frank Billings, M.D.*

The Mechanism of Indirect Fracture of the Skull. *By Charles W Dulles, M.D.*

Antisepsis in Ovariotomy and Battey's Operation. *By Robert W. Battey, M.D.*

Common Errors, Theoretical and Practical, Relating to Insanity. *By Orpheus Everts, M.D.*

Meconeuropathia. *By C. H. Hughes, M.D.*

Abstract of Proceedings of the Michigan State Board of Health, January 11, 1887.

Zur Verwendung des Sublimats bei Irrigationen in der Geburtshilfe. *Von Professor Dr. Gustav Braun.*

Zur Rückwärtslagerung des graviden Uterus. *Von Professor Dr. Gustav Braun.*

Separat-Abdruck aus der Zeitschrift für Ohrenheilkunde. *Von H Knapp und H. Moos.*

Parallelo tra il Passato ed il Presente dell'Atmiatria. *Dal Professore A. Jacobelli.*

20th

SUBSCRIPTION PRICE, $3.00 PER YEAR, IN ADVANCE.

Whole Number, 328. APRIL, 1887. Vol. LIV, Number 4.

LIBRARY INDEXED S.G.O.

THE

### CHICAGO MEDICAL JOURNAL AND EXAMINER

Editor: S. J. JONES, M.D., LL.D.

COLLABORATORS.

CHICAGO:

PROFESSOR E. ANDREWS, M.D., LL.D.

PROFESSOR J. BARTLETT, M.D.

PROFESSOR W. H. BYFORD, A.M., M.D.

PROFESSOR L. CURTIS, A.M., M.D.

PROFESSOR O. C. DEWOLF, A.M., M.D.

PROFESSOR E. C. DUDLEY, A.B., M.D.

PROFESSOR C. FENGER, M.D.

PROFESSOR ELBERT WING, A.M., M.D.

PROFESSOR J. N. HYDE, A.M., M.D.

PROFESSOR E. F. INGALS, A.M., M D.

PROFESSOR W. W. JAGGARD, A.M., M.D.

PROFESSOR F. S. JOHNSON, A.M., M.D.

PROFESSOR H. A. JOHNSON, M.D., LL.D.

PROFESSOR C. W. PURDY, M.D.

PROFESSOR C. G. SMITH, A.M., M.D.

PROFESSOR F. BILLINGS, M.D.

PROFESSOR W. T. BELFIELD, M.D.

MILWAUKEE, WISCONSIN:

PROFESSOR N. SENN, M.D.

WASHINGTON, D. C.:
S. O. RICHEY, M.D.

UNITED STATES ARMY:
SURGEON D. L. HUNTINGTON, M.D.

UNITED STATES NAVY:
MEDICAL DIRECTOR J. M. BROWNE, M.D.
MEDICAL DIRECTOR A. L. GIHON, A.M., M.D.

